# RNA editing landscape of adipose tissue in polycystic ovary syndrome provides insight into the obesity-related immune responses

**DOI:** 10.3389/fendo.2024.1379293

**Published:** 2024-06-24

**Authors:** Hanxiao Chen, Tongtong Li, Rui Gao, Meng Cheng, Qiong Zhang, Xiumei Liu, Mingli Chen, Xin Liao, Lang Qin

**Affiliations:** ^1^ Department of Obstetrics and Gynecology, West China Second University Hospital, Sichuan University, Chengdu, China; ^2^ Key Laboratory of Birth Defects and Related of Women and Children of Ministry of Education, West China Second University Hospital, Sichuan University, Chengdu, China; ^3^ Center for Reproductive Medicine, Shandong University, Jinan, Shandong, China; ^4^ Department of Physiology School of Basic Medical Sciences, Cheeloo College of Medicine Shandong University, Jinan, Shandong, China; ^5^ The Reproductive Medical Center, Department of Obstetrics and Gynecology, West China Second University Hospital, Sichuan University, Chengdu, China; ^6^ Department of Obstetrics and Gynecology, Sichuan Province Ziyang Maternal and Child Health Care Hospital, Ziyang, China; ^7^ Department of Operation Room, West China Second University Hospital, Sichuan University, Chengdu, China

**Keywords:** polycystic ovary syndrome, adipose tissue, RNA editing, inflammation, immune response

## Abstract

**Background:**

Polycystic ovary syndrome (PCOS) is the most common reproductive–endocrine disorder with wide-ranging metabolic implications, including obesity. RNA editing, a post-transcriptional modification, can fine-tune protein function and introduce heterogeneity. However, the role of RNA editing and its impact on adipose tissue function in PCOS remain poorly understood.

**Methods:**

This study aimed to comprehensively analyze RNA-editing events in abdominal and subcutaneous adipose tissue of PCOS patients and healthy controls using high-throughput whole-genome sequencing (WGS) and RNA sequencing.

**Results:**

Our results revealed that PCOS patients exhibited more RNA-editing sites, with adenosine-to-inosine (A-to-I) editing being prevalent. The expression of ADAR genes, responsible for A-to-I editing, was also higher in PCOS. Aberrant RNA-editing sites in PCOS adipose tissue was enriched in immune responses, and interleukin-12 biosynthetic process. Tumor necrosis factor (TNF) signaling, nuclear factor kappa B (NF-κB) signaling, Notch signaling, terminal uridylyl transferase 4 (*TUT4*), hook microtubule tethering protein 3 (*HOOK3*), and forkhead box O1 (*FOXO1*) were identified to be of significant differences. Differentially expressed genes (DEGs) in PCOS adipose tissue were enriched in immune responses compared with controls, and the DEGs between subcutaneous and abdominal adipose tissue were also enriched in immune responses suggesting the important role of subcutaneous adipose tissue. Furthermore, we identified the correlations between RNA editing levels and RNA expression levels of specific genes, such as ataxia–telangiectasia mutated (*ATM*) and mucosa-associated lymphoid tissue lymphoma translocation protein 1 (*MALT1*) in inflammation pathways and *ATM*, *TUT4*, and YTH N6-methyladenosine RNA-binding protein C2 (*YTHDC2*) in oocyte development pathway.

**Conclusions:**

These findings suggest that RNA-editing dysregulation in PCOS adipose tissue may contribute to inflammatory dysregulations. Understanding the interplay between RNA editing and adipose tissue function may unveil potential therapeutic targets for PCOS management. However, further research and validation are required to fully elucidate the molecular mechanisms underlying these associations.

## 1 Introduction

Polycystic ovary syndrome (PCOS) is the most common reproductive–endocrine disorder affecting reproductive-aged women worldwide (1). It is mainly characterized by three clinical manifestations, including menstrual irregularities, hyperandrogenism, and polycystic ovaries (1, 2). Recent studies have revealed that PCOS is not confined to reproductive abnormalities but also extends to metabolic disturbances, including insulin resistance, dyslipidemia, and obesity (3). Obesity is increasingly recognized as a significant comorbidity that influences the clinical manifestations and long-term health outcomes of PCOS patients (4, 5).

Adipose tissue, once considered as a mere energy storage depot, is now acknowledged as a dynamically active and metabolically influential organ (6). It exerts essential endocrine, paracrine, and autocrine functions producing adipokines, cytokines, and other bioactive molecules that regulate systemic energy homeostasis and metabolic processes (7). The association between PCOS and obesity is bidirectional, with obesity acting as both a consequence and a contributor to PCOS pathogenesis (8). In PCOS patients, dysfunction of adipose tissue contributes to the development of insulin resistance, metabolic disturbances, and chronic low-grade inflammation (9).

RNA editing is a conserved mechanism that enables specific nucleotide changes in RNA molecules, resulting in alterations in protein-coding sequences or regulatory elements (10). Adenosine-to-inosine (A-to-I) editing, catalyzed by adenosine deaminases acting on RNA (ADAR) enzymes, is the most prevalent type of RNA editing in mammals (11). RNA editing is a dynamic process that occurs within coding regions (CDSs) as well as non‐CDS regions. Notably, RNA editing has the capacity to generate start or stop codons or eliminate stop codons, thereby altering base-pairing interactions during translation and could lead to variations in amino acid sequences or modulate RNA splicing, stability, and localization (12). Consequently, RNA editing can fine-tune protein function, diversify transcript isoforms, enhance gene function diversification, and introduce heterogeneity (13). RNA editing has been observed in various organisms, tissues, and developmental stages indicating its widespread biological importance. Aberrant RNA-editing events were associated with numerous human diseases, including cancer (14), metabolic disorders (15), and immune responses (16). However, the role of RNA editing in PCOS and its impact on adipose tissue function remain poorly understood.

Understanding the landscape of RNA editing in adipose tissue from PCOS patients may provide valuable insights into the molecular mechanisms underlying the metabolic abnormalities observed in this syndrome. By identifying specific RNA-editing events and their associated target genes, we can gain a deeper understanding of the functional consequences of RNA-editing dysregulation in PCOS adipose tissue. Furthermore, unraveling the interplay between RNA editing and adipose tissue dysfunction may reveal potential therapeutic targets for the clinical management of PCOS.

In this study, we aimed to conduct a comprehensive analysis of RNA-editing events in abdominal and subcutaneous adipose tissue derived from PCOS patients and healthy controls. Utilizing high-throughput whole-genome sequencing (WGS) coupled with RNA sequencing, we identified differentially edited RNA transcripts and characterized their functional impacts on adipose tissue metabolism and inflammation. Additionally, we also explored the expression patterns and activities of ADAR enzymes to gain insights into the regulation of RNA editing in PCOS adipose tissue.

## 2 Materials and methods

### 2.1 Study population and sample collection

PCOS patients who underwent laparoscopic surgery for hydrosalpinx at the Department of Obstetrics and Gynecology, West China Second University Hospital, from January 2022 to February 2022, were enrolled in this study. Patients were diagnosed with PCOS according to the criteria of the European Society for Human Reproduction and Embryology/American Society for Reproductive Medicine (ESHRE/ASRM) (Rotterdam criteria) (2). Two or more of the following three criteria should be met including hyperandrogenism, oligo-/anovulation, and polycystic ovaries on transvaginal ultrasound (TVS). Patients with any known significant disorders that may overlap or confound the diagnosis of PCOS, such as congenital adrenal hyperplasia, functional hypothalamic amenorrhea (FHA), Cushing syndrome, thyroid diseases, hyperandrogenism due to non-PCOS causes, androgen-secreting ovarian tumors or adrenal tumors, and premature ovarian insufficiency (POI), were excluded. Patients who underwent laparoscopic surgery for hydrosalpinx without any clinical manifestation of PCOS were enrolled as the control group. Abdominal adipose tissue and subcutaneous adipose tissue were obtained, and samples were stored at −80°C until use. The Ethical Review Board of West China Second University Hospital, Sichuan University, approved this study [Approval number: No. 2021(033)].

### 2.2 RNA sequencing

The RNA‐seq dataset for abdominal adipose tissue and subcutaneous adipose tissue from the PCOS and control patients was uploaded to the Gene Expression Omnibus (GEO) database with accession number PRJNA1010231. Total RNA was harvested using TRIzol reagent (Invitrogen, Carlsbad, CA, USA) following the instructions provided by the manufacturer. Total amounts and integrity of RNA were assessed using the RNA Nano 6000 Assay Kit of the Bioanalyzer 2100 system (Agilent Technologies, CA, USA). RNA libraries were prepared for sequencing using the NEBNext^®^ Ultra RNA Library Prep Kit for Illumina^®^ (NEB, USA). Illumina NovaSeq 6000 was used, and the qualified libraries were pooled and sequenced on Illumina platforms with the PE150 strategy of Novogene Bioinformatics Technology Co., Ltd. (Beijing, China). Raw data of fastq format were first processed through in-house perl scripts. Then, we used the following parameters to trim the data: fastp -n 15 -q 5 -u 50 -l 150. Reads containing adapter, reads containing N base, and low-quality reads from raw data were removed to obtain clean data. Concurrently, Q20, Q30, and GC content clean data were calculated, and all of the downstream analyses were based on the high-quality clean data. Paired-end clean reads were mapped to the reference human genome (GRCh38) using Hisat2 (version 2.0.5) (http://github.com/infphilo/hisat2), and alignment files were generated in Binary Alignment Map (BAM) format. To quantify the gene expression level, featureCounts (version 1.5.0-p3) was adopted to count the read numbers mapped to each gene. Then, FPKM, expected number of Fragments Per Kilobase of transcript sequence per Millions base pairs sequenced, of each gene was calculated based on the length of the gene, and read counts were mapped to this gene.

### 2.3 Whole-genome sequencing

The WGS dataset for abdominal adipose tissue and subcutaneous adipose tissue from the PCOS and control patients was uploaded to the GEO database with accession number PRJNA1010231. Genomic DNA was extracted from the adipose samples using the standard phenol–chloroform protocol. DNA libraries were generated using the NEB Next^®^ Ultra™ DNA Library Prep Kit for Illumina (NEB, USA) according to the manufacturer’s instructions. DNA concentration was measured using Qubit^®^ DNA Assay Kit in Qubit^®^ 3.0 Flurometer (Invitrogen, USA). The sequencing libraries were analyzed for size distribution using NGS3K/Caliper and quantified using real-time PCR (3 nM). Clustering of the index-coded samples was performed on a cBot Cluster Generation System using Illumina PE Cluster Kit (Illumina, USA). Illumina NovaSeq 6000 was used, and the qualified libraries were pooled and sequenced on Illumina platforms with the PE150 strategy of Novogene Bioinformatics Technology Co., Ltd. (Beijing, China). We used Fastp (version 0.19.7) to perform quality control analysis of the generated raw data. A paired read was discarded if either one read contains adapter contamination, more than 10% of bases are uncertain in either one read, or if the proportion of low quality (Phredquality <5) bases is over 50% in either one read. Sequencing data is mapped to the reference human genome (GRCh38), alignment files in BAM format were obtained, and VCF files were generated by an intelligent web portal (GTX.Digest system) for genomics data interpretation (17). The filter parameters of single-nucleotide variations (SNVs) are shown as follows: genotype quality score ≥30; gene quality score ≥2.

### 2.4 Identification and annotation of RNA-editing candidates

We employed REDItools software for detecting candidate sites (18). Subsequently, mismatches between the initial reads and the reference genome were collected. Multiple test correction was conducted to adjust the p-value using the p.adjust function in R, and RNA-editing sites with a false discovery rate >0.05 were removed. RNA editing was annotated using the Annovar software (19). The Ensembl and National Center for Biotechnology Information (NCBI) databases (Homo_sapiens.GRCh38.100.gtf) provided the reference gene set for annotation. We also extracted the variants with A/G and C/T mismatch or T/C and G/A mismatch corresponding to plus and minus strand (20). The RNA-editing events with an alternative allele frequency (AAF) of 1% or higher and less than 100% were included. To eliminate SNPs, we download the SNP data from the dbSNP database (https://ftp.ncbi.nih.gov/snp/organisms/human_9606/BED/) and Ensembl database (https://ftp.ensembl.org/pub/release-111/variation/vcf/homo_sapiens/), and bedtools was used to remove all SNPs in our data from the databases. We also compared the detected RNA-editing sites with the known human RNA-editing sites from the REDIportal V2.0 database. Repeat sequences were obtained from the University of California, Santa Cruz (http://genome.ucsc.edu). Alu sequences were extracted using a Python program, and subsequently, the editing sites within the Alu sequences were analyzed (21). Visualization of RNA-editing sites of PCOS and control groups in different chromosomes was performed using the online web tool shinyCircos-V2.0 (https://venyao.xyz/shinycircos/) (22, 23).

### 2.5 Essential and nonessential genes

We obtained essential (Ess) and nonessential (Noness) genes of humans from the OGEE (Online GEne Essentiality) database (https://v3.ogee.info/static/files/gene_essentiality.txt.gz) (24). We investigated the effect of gene essentiality of the detected RNA-editing genes because essential genes are those that are functionally critical for the survival of an organism or cell and capture the intricate nature of biological systems. Building upon this distinction, we discovered substantial differences in the numbers of edited genes between Ess and Noness categories.

### 2.6 Integrative Genome Viewer analysis

In this study, we utilized the bamCoverage function of deeptools (version 3.5.5) to convert aligned bam files into bw files, therefore facilitating visualization of gene expression abundance in the integrative genomics viewer (IGV) (version 2.17.4) (http://www.broadinstitute.org/software/igv/download) (25). Additionally, we loaded bam files from WGS and RNA-seq to display the read mapping results across the entire genome and examined the RNA-editing sites of genes.

### 2.7 Partial least squares-discriminant analysis

To illustrate and cluster the potential differences between PCOS and control groups, we adopted the partial least squares-discriminant analysis (PLS-DA) method (26). PLS-DA is a supervised dimensionality reduction method and commonly used in metabolomics since it can more effectively highlight metabolites that differentiate between conditions. R package ropls (version 1.34.0) was used to perform PLS-DA, and R package ggplot2 (version 3.3.6) was used to visualize the result.

### 2.8 Differential expression analysis

The DESeq2 R package (version 1.20.0) (27) was utilized to conduct differential expression analysis among distinct groups. DESeq2 offers statistical routines to ascertain differential expression in digital gene expression data by employing a model grounded on the negative binomial distribution. To control the false discovery rate (FDR), the raw p-values underwent adjustment using the Benjamini–Hochberg (BH) method. The threshold for significantly differentially expressed genes was established as p.adjust ≤ 0.05 and |log2(foldchange)| ≥ 1.

### 2.9 Functional enrichment analysis

The functional enrichment analysis was conducted using R package clusterProfiler (version 3.16.0) (28) to identify significantly enriched Gene Ontology (GO) biological processes terms. We not only performed GO analysis on the deferential RNA-editing sites but also on the differentially expressed genes between the abdominal or subcutaneous adipose tissue of PCOS and control patients. The BH method was employed for adjusting the p-value to identify significantly enriched GO biological processes terms, and terms with p.adjust <0.05 were considered significantly enriched.

### 2.10 Statistical analysis

Wilcoxon tests or Kruskal–Wallis tests were used in identifying differential RNA-editing sites and genes and RNA editing frequencies between two or more groups. The χ2 and F-tests were used as the statistical analysis for categorical variable, such as proportion of essential and nonessential genes. A value of p < 0.05 was used as the significance cutoff. For gene expression data, raw p-values were adjusted using the BH method, and adjusted p < 0.05 was considered significant in detecting functional enriched pathways with differentially edited or differentially expressed genes. The association between RNA editing level and gene expression level was calculated using Spearman’s rank correlation to obtain the correlation coefficient (r) and p-value (29). Statistical analyses were performed using R or GraphPad Prism 9 software.

## 3 Results

### 3.1 PCOS patients have more editing sites than healthy women

**Figure 1:**
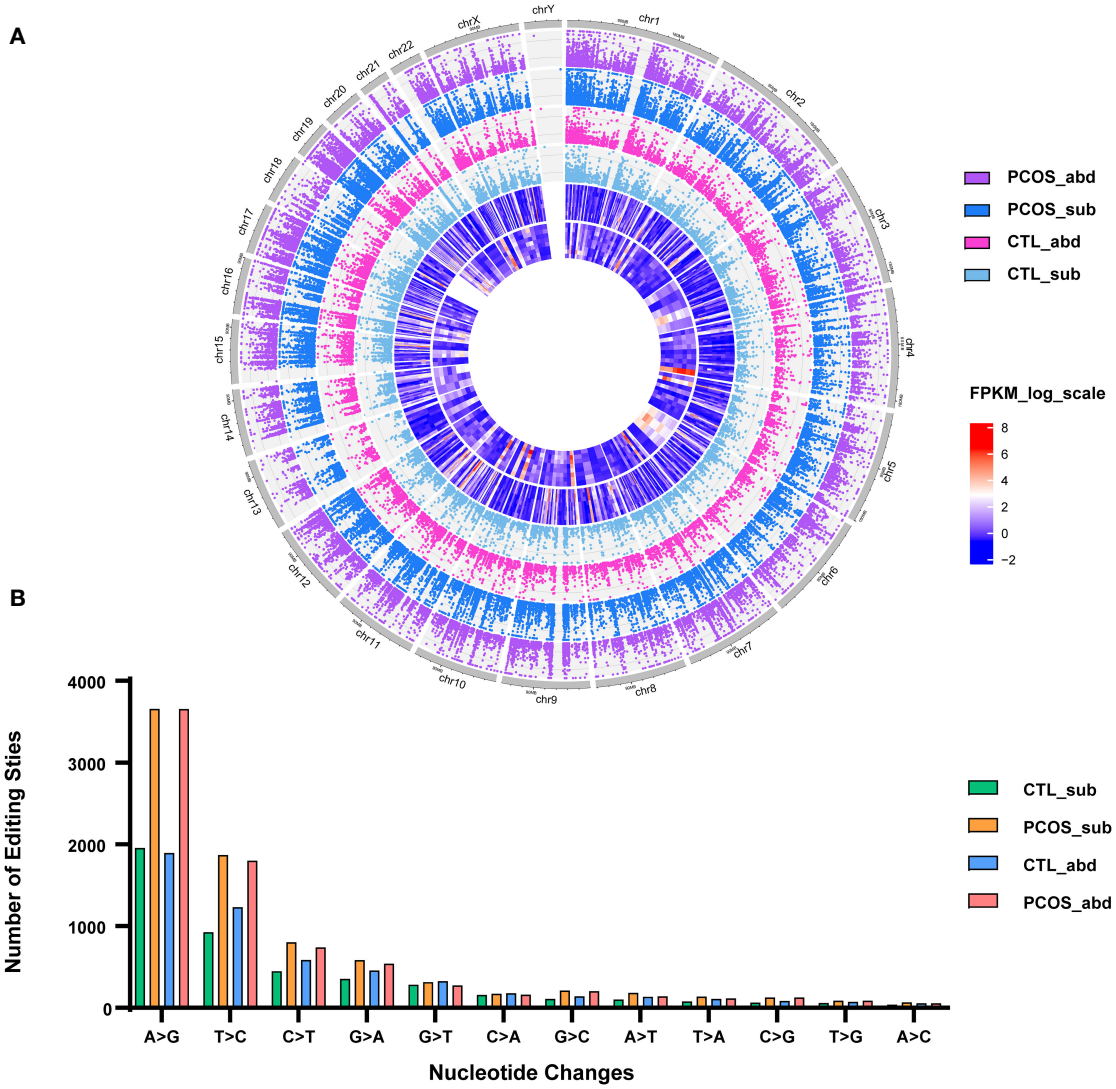
Distributions of RNA-editing events in abdominal and subcutaneous adipose tissue of PCOS and control patients. **(A)** Circos plot shows the distributions of RNA-editing events in different chromosomes. The dots represent the RNA editing levels of each RNA-editing site. Purple represents the abdominal adipose tissue of PCOS, blue represents the subcutaneous adipose tissue of PCOS, pink represents the abdominal adipose tissue of control, light blue represents the subcutaneous adipose tissue of the control. The outer heatmap shows the RNA expression levels of differentially expressed genes between the abdominal adipose tissue of the control and PCOS patients. The inner heatmap shows the RNA expression levels of differentially expressed genes between the subcutaneous adipose tissue of the control and PCOS patients. **(B)** Distribution of RNA-editing types. The *x*-axis is 12‐nucleotide changes, and the *y*-axis is the number of edits. *p-value <0.05.

This study enrolled five PCOS patients and three control patients (**Supplementary Table S1**). Paired subcutaneous and abdominal adipose tissues were collected and used for WGS and RNA sequencing, and RNA-editing sites were identified (**Supplementary Figures S1A, B**). In subcutaneous adipose tissue of PCOS patients, an average of 8,213 RNA-editing events and 3,387 RNA-editing genes were identified, 7,898 sites and 3,329 genes were edited in the abdominal adipose tissue of PCOS, 4,575 sites and 2,366 genes in the subcutaneous adipose tissue of the control, and 5,267 sites and 2,911 genes in the abdominal adipose tissue of the control (**Supplementary Figures S2A, B**). Compared with the healthy controls, we found more RNA-editing sites and edited genes in the subcutaneous and abdominal adipose tissue of PCOS patients, but the differences in editing genes did not reach statistical significance. In addition, we found that approximately half of our detected RNA-editing sites match those already cataloged in the REDIportal V2.0 database (**Supplementary Figure S2C**). Circos plot was drawn to show the distributions of RNA-editing events in different chromosomes of different groups (**Figure 1A**). As shown in the figure, chromosome 1 contains the most RNA-editing events because it is the longest and, therefore, contains genes encoding the largest number of transcripts capable of undergoing RNA editing. The main types of nucleotide substitutions in humans are A‐to‐I and uridine‐to‐cytidine (U‐to‐C) followed by cytidine‐to‐uridine (C‐to‐U) and guanine-to-adenosine (G-to-A) (**Figure 1B**).

**Figure 2:**
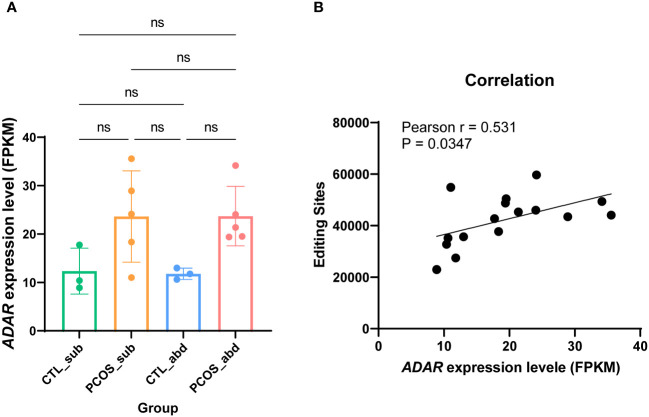
The relationship between ADAR genes and RNA-editing sites. **(A)** Expression levels of ADAR genes across the abdominal and subcutaneous adipose tissues of PCOS and control patients. **(B)** The relationship between ADAR genes and RNA-editing sites among the samples. ns means p>0.05.

It is well known that A-to-I editing is catalyzed by ADAR enzymes (30). Hence, we investigated whether the differences in RNA editing are related to the differential expression of the ADAR genes. First, we calculated the expression levels of the ADAR gene using the RNA-seq data. The total expression of the ADARs is higher in PCOS than in the control, although it did not reach statistical significance (**Figure 2A**). Statistically significant correlations were also observed for ADAR gene expression and the number of editing sites (r = 0.531, p = 0.0347) (**Figure 2B**).

### 3.2 Functional distribution of editing sites

**Figure 3:**
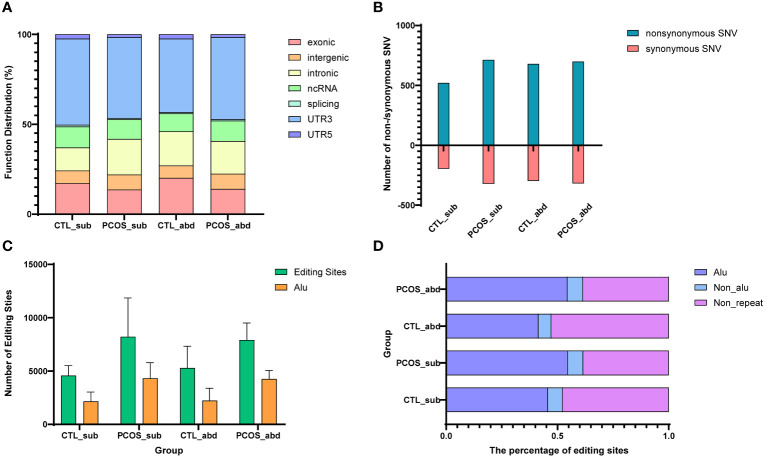
Distribution of RNA-editing functional regions and editing level trends. **(A)** Function distribution of RNA-editing sites in abdominal and subcutaneous adipose tissues of PCOS and control patients. **(B)** The number of nonsynonymous and synonymous editing in CDSs. Blue represents the number of nonsynonymous edits, and red represents the number of synonymous edits. **(C)** Distribution of the Alu region and number of editing sites in the abdominal and subcutaneous adipose tissues of PCOS and control patients. Yellow represents the number of editing sites occurring in the Alu region, and green represents the number of all RNA-editing sites. **(D)** The percentage of editing sites in the repeat sequence for humans. Blue represents the proportion of the editing sites occurring in the Alu region, cyan represents the repeating region except Alu, and purple represents the non-repeating region.

In this study, we found that RNA editing in adipose tissue mainly occurs in UTR3, exonic regions, introns, and noncoding regions (**Figure 3A**). Our findings demonstrated that nonsynonymous editing was more than synonymous editing in the adipose tissue of the PCOS and healthy control (all p < 0.05) (**Figure 3B**). These findings indicated that RNA editing may play an important role in the adipose tissue of PCOS. We also found that the number of RNA-editing events in the Alu regions in PCOS remains higher than in the control probably due to more total RNA-editing sites in PCOS than in the control (**Figure 3C**). In addition, the proportion of RNA-editing events in the Alu repeating region is also higher in the subcutaneous and abdominal adipose tissues of PCOS than in the control (**Figure 3D**).

Next, we identified the Ess and Noness genes of humans from the OGEE database. Ess genes are crucial for the survival of organisms, whereas Noness genes are not indispensable and rely on external conditions rather than intrinsic properties for their necessities. Then, we counted the Ess and Noness genes at the editing site of the CDS, and our results demonstrated that the number of Noness genes among all editing genes or nonsynonymous editing genes in humans is significantly higher than the Ess genes in all groups (all p < 0.05) (**Supplementary Figures S3A, B**). The higher number of editing sites in Noness genes is due to the fact that there are typically more Noness genes than Ess genes. To explore whether these nonsynonymous editing genes differ in the necessities of genes, we compared the editing frequency of Ess and Noness genes in the nonsynonymous editing genes. There was no significant difference in the editing frequency of Ess and Noness genes in all groups (all p > 0.05 in **Supplementary Figure S3C**).

We also investigated the RNA editing level, also known as RNA editing frequency, in abdominal and subcutaneous adipose tissues of PCOS and control patients. There were no significant differences in RNA editing level between abdominal and subcutaneous adipose tissues of PCOS and control patients (**Supplementary Figure S4A**). Our findings revealed a slightly higher editing frequency of nonsynonymous genes than synonymous genes in subcutaneous adipose tissue of the PCOS group (**Supplementary Figure S4B**). In summary, we verified that RNA editing tends to present in Noness genes and that similar results exist in both abdominal and subcutaneous adipose tissues of PCOS and control patients.

### 3.3 Differentially edited genes between adipose tissue of PCOS and control patients

**Figure 4:**
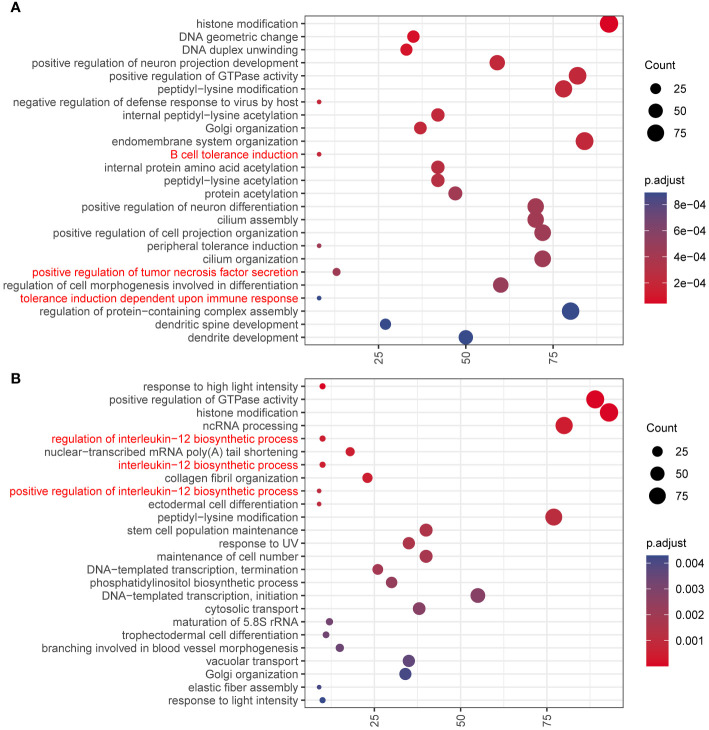
GO enrichment analysis of the genes containing PCOS-specific RNA editing. Dot plot of the enriched GO Biological Progress terms of genes **(A)** only edited in the abdominal adipose tissue of PCOS but not in the control patients and **(B)** only edited in the subcutaneous adipose tissue of PCOS but not in the control patients. Dot color indicates the statistical significance of the enrichment (adjusted p-value); dot size represents the count of genes annotated to each term. Red represents the terms related to immune and inflammatory processes.

It is also found that RNA editing differs in different stages, chromosomes, and editing types. In humans, RNA-editing sites are concentrated on chromosome 1 (**Figure 1C**). We explored the genes with nonsynonymous RNA editing and performed function enrichment analysis. First, Venn plots was drawn to identify tissue or PCOS-specific RNA-edited genes (**Supplementary Table S2** and **Supplementary Figure S5**). We found that only 2,034 genes in the abdominal adipose tissue of PCOS, but not in the control patients, were mainly enriched in immune responses, including B-cell tolerance induction, positive regulation of tumor necrosis factor (TNF) secretion, and tolerance induction dependent upon immune response pathways (**Figure 4A**). More specifically, major histocompatibility complex, class I, G (*HLA-G*), leukocyte immunoglobulin-like receptor A5 (*LILRA5*), leucine-rich repeat kinase 2 (*LRRK2*), Wnt family member 5A (*WNT5A*), frizzled class receptor 5 (*FZD5*), and *LILRA2* genes were only edited in the abdominal adipose tissue of PCOS but not in the control. Similarly, only 2,077 genes edited in the subcutaneous adipose tissue of PCOS, but not in the control patients, were mainly enriched in interleukin-12 (IL-12) biosynthetic process and stem cell population maintenance (**Figure 4B**). Genes enriched in inflammation, such as lymphotoxin beta (*LTB*), TNF receptor-associated factor 6 (*TRAF6*), and nuclear factor kappa B subunit 1 (*NFKB1*), and genes enriched in stem cell population maintenance, such as delta-like canonical Notch ligand 1 (*DLL1*), notch receptor 1 (*NOTCH1*), *NOTCH2*, replication timing regulatory factor 1 (*RIF1*), *FZD7*, terminal uridylyl transferase 4 (*TUT4*), hook microtubule-tethering protein 3 (*HOOK3*), DNA ligase 4 (*LIG4*), forkhead box P1 (*FOXP1*), and forkhead box O1 (*FOXO1*), were merely edited in the subcutaneous adipose tissue of PCOS. In addition, genes differently edited between the abdominal and subcutaneous adipose tissues of PCOS patients were also enriched in immune processes (**Supplementary Figure S6**).

### 3.4 Differentially expressed genes between adipose tissue of PCOS and control patients

**Figure 5:**
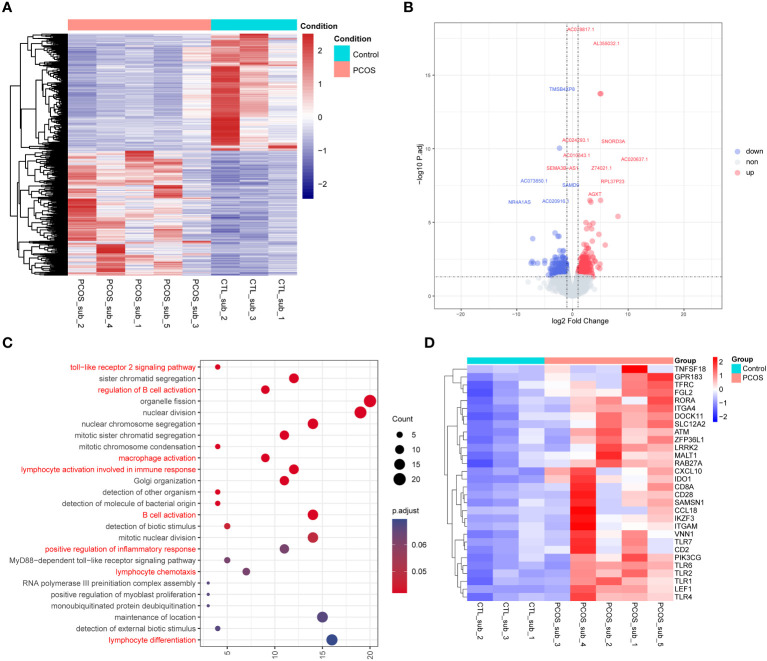
RNA sequencing of the subcutaneous adipose tissue of PCOS and control patients. **(A)** Heatmap of the differentially expressed genes. **(B)** Volcano plot of the differentially expressed genes. Blue represents downregulated-expression genes, and red represents upregulated-expression genes. **(C)** Dot plot of the enriched GO Biological Progress terms of the upregulated-expression genes in the subcutaneous adipose tissue of PCOS compared with the control group. Dot color indicates the statistical significance of the enrichment (adjusted p-value); dot size represents the count of genes annotated to each term. Red represents the terms related to immune and inflammatory processes. **(D)** Heatmap of the differentially expressed genes enriched in the immune-related genes between the subcutaneous adipose tissues of PCOS and control patients.

RNA sequencing data were also analyzed to investigate the differentially expressed genes between the adipose tissue of PCOS and control patients. PLS-DA plot and heatmap of the cluster analysis of different samples revealed great homogeneity within groups (**Supplementary Figure S7**). A total of 1,030 differentially expressed genes were identified between the subcutaneous adipose tissue of PCOS and control patients (**Supplementary Table S3** and **Figures 5A, B**). GO enrichment analysis revealed that genes differentially expressed between the subcutaneous adipose tissue of PCOS and control patients were mainly involved in the immune responses such as Toll-like receptor 2 signaling pathway, regulation of B-cell activation, macrophage activation, lymphocyte activation involved in immune response, positive regulation of inflammatory response, and lymphocyte differentiation (**Figure 5C**). This is in accordance with our previous findings that genes only edited in the subcutaneous adipose tissue of PCOS but not in control patients were mainly enriched in immune responses (**Figure 4B**). In addition, we also drew a heatmap for the differentially expressed immune-related genes (**Figure 5D**).

We also identified 4,306 differentially expressed genes between abdominal adipose tissue of PCOS and control patients. However, instead of immune processes, genes differentially expressed between the abdominal adipose tissue of the PCOS and control patients were mostly enriched in signal transduction, cell structure organization, metabolic process, etc. (**Supplementary Table S4** and **Supplementary Figure S8**). We also explore the differences between the abdominal and subcutaneous adipose tissue of PCOS patients (**Supplementary Table S5** and **Supplementary Figure S9A**). The upregulated genes in the subcutaneous adipose tissue of PCOS, compared with the abdominal adipose tissue of PCOS, were also mainly enriched in immune responses (**Supplementary Figure S9B**), and the downregulated genes were enriched in cellular process, cell death, etc. (**Supplementary Figure S9C**). Additionally, some known adipokine genes, such as chemerin-encoding gene retinoic acid receptor responder 2 (*RARRES2*), were also detected in our datasets (**Supplementary Tables S3** and **S4**).

### 3.5 The association between differentially edited genes and differentially expressed genes

Finally, we aimed to study the relationship between RNA editing level and RNA expression level of the differentially expressed genes and focused on genes of inflammation and immune response pathways. Our results found that there was a positive correlation between RNA editing level and RNA expression level in some genes, such as ataxia–telangiectasia mutated (*ATM*) (r = 0.53, p = 0.036), CD8 subunit alpha (*CD8A*) (r = 0.76, p = 0.00067), lymphoid enhancer-binding factor 1 (*LEF1*) (r = 0.55, p = 0.027), mucosa-associated lymphoid tissue lymphoma translocation protein 1 (*MALT1*) (r = 0.56, p = 0.025), and Toll-like receptor 7 (*TLR7*) (r = 0.51, p = 0.046) (**Supplementary Figure S10**). Next, we used IGV software to visualize these RNA-editing sites and the same regions covered by DNA-seq data, such as *ATM* and *MALT1* (**Supplementary Figure S11**). Our data revealed RNA-editing events occurring at position chr18:58,753,294 in *MALT1*, and the RNA-seq results also showed significant changes in expression levels near this site. Moreover, the editing level at this site was notably higher in PCOS patients than those in normal controls. However, further experimental validation is required to determine whether this editing event can actually influence gene expression.

**Figure 6:**
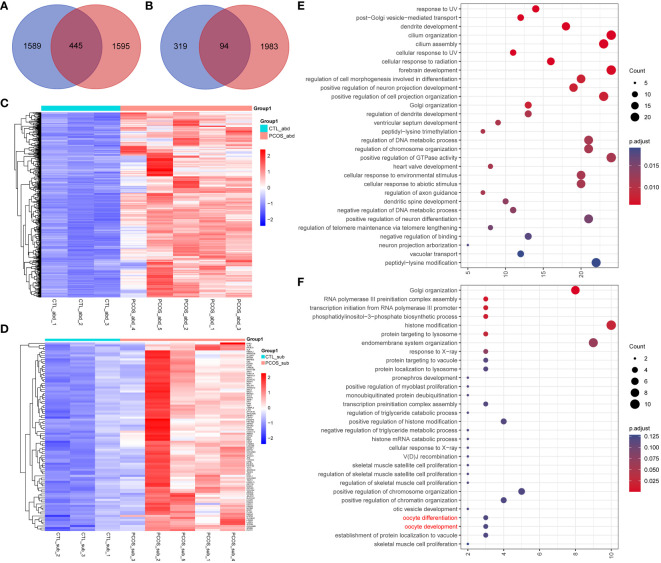
The association between differentially edited genes and differentially expressed genes. **(A, B)** Venn plot represents the intersection of genes only edited in **(A)** abdominal and **(B)** subcutaneous adipose tissues of PCOS and differentially expressed genes between PCOS and the control group. **(C, D)** Heatmap of the intersected genes in **(C)** abdominal and **(D)** subcutaneous adipose tissue of PCOS and the control group. **(E, F)** Dot plot of the enriched GO Biological Progress terms of the intersected genes in **(E)** abdominal and **(F)** subcutaneous adipose tissue.

Moreover, we also looked into all the genes that are both differentially edited and differentially expressed between the PCOS and control groups. Our results indicated that 445 differentially expressed genes are exclusively edited in the abdominal adipose tissue of PCOS, and 94 differentially expressed genes are only edited in the subcutaneous adipose tissue of PCOS (**Figures 6A, B**). Heatmaps were drawn to directly visualize the RNA expression level of differentially expressed genes that were exclusively edited in PCOS (**Figures 6C, D**). Interestingly, using functional enrichment analysis (**Figures 6E, F**), we found three subcutaneous PCOS-specific edited differentially expressed genes, *ATM*, *TUT4*, and YTH N6-methyladenosine RNA-binding protein C2 (*YTHDC2*), enriched in the oocyte development and differentiation pathway, which may also indicate the important role of adipose tissue in the pathogenesis of PCOS.

## 4 Discussion

In this research, we performed WGS and RNA sequencing to investigate the RNA-editing events in the abdominal and subcutaneous adipose tissue derived from PCOS patients and healthy controls. Our analysis of differentially edited and expressed genes suggests that RNA-editing events in PCOS adipose tissue are associated with immunological alterations offering novel insights into PCOS pathogenesis and providing avenues for future investigation into the biological functions of adipose tissue in PCOS.

RNA editing, a post-transcriptional modification process, has been reported to contribute to the dysregulation of gene expression and is involved in cancer, metabolic disorders, and immune responses (31), yet its role in PCOS is still not well understood. One previous article performed RNA-editing analysis and used RNA-seq data of granulosa cells (GCs) of PCOS from public databases. They suggested a potential role of RNA editing in the pathophysiology of PCOS (32). However, PCOS is a highly heterogenous syndrome, and the dysfunction of adipose tissue in PCOS is likely involved in the pathogenesis of this syndrome and associated metabolic disturbances. Therefore, in this study, we focused on the RNA-editing events in the adipose tissue of PCOS and observed more editing events in the adipose tissue of PCOS patients compared to the healthy controls, regardless of whether it was subcutaneous or abdominal adipose tissue. The RNA-editing events were mainly located in the UTR3, exonic regions, and introns, and the distributions were similar between the PCOS and control groups. Our results revealed more nonsynonymous editing than synonymous editing in all groups. The overall RNA editing frequency was similar between each group. Our findings suggest that the A‐to‐I was the predominant RNA editing pattern, and we also found that compared with the healthy controls, PCOS patients had more A‐to‐I RNA-editing sites in the adipose tissue. To understand the reasons behind these disparities, we explored the expression of the ADAR genes and its association with RNA-editing sites since ADAR catalyzes the posttranscriptional conversion of A-to-I in double-stranded RNA (dsRNA) (30). Our results revealed a higher expression of the ADAR gene in the PCOS groups compared with the control groups, and ADAR gene expression was positively correlated with the number of RNA-editing sites. We also investigated the RNA editing in Alu repeat region sequence, which is a major prerequisite for most A‐to‐I RNA editing (33). In addition, we found that, compared with the adipose tissue of the control group, the PCOS groups had more RNA-editing sites in the Alu repeat region. Therefore, our findings suggest the important role of ADAR enzyme and its mediated A‐to‐I RNA editing in the pathogenesis of PCOS.

Then, we investigated the PCOS-specific RNA-editing genes. PCOS-specific editing events in abdominal adipose tissue were enriched in immune response tolerance induction and regulation of TNF secretion, and we also identified specific genes such as *HLA-G* and *WNT5A*. Previous studies have identified the relationship between serum HLA-G level and high-density lipoprotein cholesterol, insulin resistance, ovarian hyperandrogenism, and oxidative stress in women with PCOS (34). Another study showed that the upregulated expression of *WNT5A* in PCOS could induce inflammation and oxidative stress through the phosphatidylinositol 3-kinase (PI3K)/AKT/nuclear factor-κB (NF-κB) signaling pathway (35). In addition, PCOS-specific editing events in subcutaneous adipose tissue were enriched in IL-12 biosynthetic process and stem cell population maintenance, and *TRAF6*, *NFKB1*, *DLL1*, *NOTCH1*, *NOTCH2*, *TUT4*, *HOOK3*, and *FOXO1* were identified as important differential edited genes. These results were consistent with previous studies that obesity could induce chronic low-grade inflammation in PCOS patients (36). One previous study showed that IL-12 and TNFα levels in the plasma were increased in PCOS patients compared to controls (37), and another study revealed the correlation between follicular fluid IL-12 levels and increased T-lymphocyte numbers in the PCOS patients (38). TNF signaling is known to have an essential role in the pathogenesis of PCOS. Women with PCOS exhibit elevated serum concentrations of TNF and C-reactive protein (CRP), along with increased levels of monocytes and lymphocytes, and obesity and hyperinsulinemia could further exacerbate the chronic inflammatory condition (39). A well-recognized function of NF-κB signaling is regulation of inflammatory responses. Therapies targeting NF-κB pathway could reduce inflammation, cell apoptosis, and autophagy. For example, one studies showed that miR-93–5p could induce apoptosis and ferroptosis in GCs by modulating the NF-κB signaling pathway (40). Notch signaling is reported to be activated in GCs in PCOS and regulates aberrant cumulus–oocyte complex (COC) expansion, which may lead to ovulatory dysfunction (41). It was also involved in adipose tissue inflammation and metabolic dysregulation (42). *TUT4* belongs to RNA terminal uridyltransferases, and it has been suggested to play a role in post-transcriptional regulation of inflammation based on *in vitro* studies (43). *HOOK3* phosphorylation can drive modulate Golgi stability, and *HOOK3* was also identified by the other RNA-editing study of GCs of PCOS (32), suggesting that RNA-editing events at this gene region is of great importance in PCOS. *FOXO1* is known to be involved in stem cell differentiation and inflammation (44–46). A recent study found that inhibition of *FOXO1* alleviates the pathological changes and ovarian dysfunction in PCOS rat models by inhibiting the TLR4/NF-κB/NLRP3 pathway to alleviate inflammation and immune responses (47). Therefore, our findings indicate distinct RNA-editing events in adipose tissue functions among PCOS patients compared to healthy controls suggesting a potential link between adipose tissue dysfunction and inflammation. Future clinical and experimental studies are needed to validate the clinical significance of such RNA editing and explore the therapeutic potentials of these gene sites.

Furthermore, comparing the difference between PCOS and control patients, we found 1,030 differentially expressed genes in subcutaneous adipose tissue and 4,306 genes in abdominal adipose tissue. Functional enrichment analyses suggested Toll-like receptor 2 signaling pathway, B-cell activation, macrophage activation, lymphocyte activation, inflammatory response, and lymphocyte differentiation pathways. We also compared the differences between the subcutaneous and abdominal adipose tissues in PCOS to ascertain whether the inflammation is caused by subcutaneous or abdominal adipose tissue. It turned out that the immune and inflammation pathways were enriched in the subcutaneous adipose tissue of PCOS. But previous research mainly focused on the visceral adipose tissue, and some suggested that visceral adiposity index plays an important role in predicting clinical severity and therapeutic outcome of women with PCOS (48, 49). One study found an increased level of IL-6 only in the visceral adipose tissue of PCOS rats, and TNF-α and IL-6 in the subcutaneous adipose tissues of PCOS rats were similar to those of the controls (50), while another study suggested that increased subcutaneous adipose tissue could lead to hyperandrogenism and enlarged visceral adipose tissue volume in PCOS patients (51). In this study, we also proposed that subcutaneous adipose tissue also plays a significant role in PCOS pathogenesis, and its alterations may contribute to the development of PCOS-related chronic low-grade inflammation.

Finally, we also investigated the association between differentially edited genes and differentially expressed genes in this study. We focused on the inflammation-related genes and found a positive correlation between RNA editing level and RNA expression level in the *ATM* and *MALT1* genes. We also identified three subcutaneous PCOS-specific edited differentially expressed genes, *ATM*, *TUT4*, and *YTHDC2* enriched in the oocyte development pathway. *ATM* is a serine/threonine protein kinase with an important regulatory function in the DNA damage response. One study indicated that persistent DNA damage-driven autoinflammation plays an essential role in adipose tissue degeneration, and this response requires *ATM* (52). In addition, *ATM*-mediated DNA double-strand break repair mechanisms, in general, are also related to ovarian aging (53, 54). A previous study found that *MALT1* was positively correlated with Th17 cells, and inflammation and its decrement, along with treatment, reflects the response to TNF inhibitor in ankylosing spondylitis patients (55). Therefore, it could be a potential biomarker for the treatment of chronic inflammation in PCOS patients. A recent study indicated that *YTHDC2* regulates meiosis in humans, and pathogenic variants of this gene are associated with POI (56). Future clinical and experimental studies are needed to determine whether these RNA-editing event can lead to alterations in gene expression and PCOS phenotypes.

Our study has several advantages. First, the comprehensive analysis conducted through WGS and RNA sequencing allows for a detailed exploration of RNA editing patterns, and our results enhance the understanding of the molecular mechanisms underlying PCOS and its potential implications for adipose tissue metabolism and inflammation. Additionally, the investigation of ADAR enzymes’ expression patterns and activities provides valuable insights into the regulatory mechanisms governing RNA editing in PCOS adipose tissue. However, there are also several limitations to consider. First, we were only able to obtain three biological replicates for the control group, which could at least meet the minimum standard in this type of research. Second, this study only collected the adipose tissue of the PCOS patients instead of their ovarian tissue due to the ethical constraints. This may limit the comprehensive understanding of the interactions between the reproductivity–metabolism–immune network in the pathogenesis of PCOS. Future studies enrolling more participants are needed. Also, the complexity of RNA-editing processes may pose challenges in interpreting functional impacts accurately potentially requiring further experimental validation to substantiate the predicted RNA-editing effects in *in vitro* and *in vivo* PCOS models.

## 5 Conclusions

In summary, our study provides an RNA-editing landscape of PCOS adipose tissue, and we also investigated the functional implications of differentially edited or expressed genes. Our discoveries revealed higher ADAR expression level and the resulting more RNA-editing events in the adipose tissue of PCOS patients. These RNA-editing events of the adipose tissue could affect the gene expression and eventually contribute to the immune and inflammatory processes. These findings could pave the way for future research and therapeutic interventions targeting RNA editing as a potential avenue for understanding and treating PCOS-related metabolic and inflammatory dysregulations.

## Data availability statement

The datasets presented in this study can be found in online repositories. The names of the repository/repositories and accession number(s) can be found below: https://www.ncbi.nlm.nih.gov/bioproject/PRJNA1010231/.

## Ethics statement

The studies involving humans were approved by The Ethical Review Board of West China Second University Hospital, Sichuan University. The studies were conducted in accordance with the local legislation and institutional requirements. The participants provided their written informed consent to participate in this study.

## Author contributions

HC: Conceptualization, Data curation, Formal Analysis, Writing – original draft, Writing – review & editing. TL: Data curation, Formal Analysis, Writing – review & editing. RG: Formal Analysis, Writing – review & editing. MeC: Software, Writing – review & editing. QZ: Software, Writing – review & editing. XL: Visualization, Writing – review & editing. MiC: Validation, Writing – review & editing. XL: Conceptualization, Writing – review & editing. LQ: Conceptualization, Funding acquisition, Writing – review & editing.

## Supplementary material

Supplementary Figure 1 Mapping of the RNA editing sites and the DNA sequencing data of each individual visualizing by Circos plot. **(A)** control samples. From left to right is control 1 to 3, respectively. **(B)** PCOS samples. From left to right and up to down is PCOS 1 to 5, respectively. Pink dots represent the quality score of each SNV from DNA sequencing data. Orange dots represent the RNA editing levels of each RNA editing sites in abdominal adipose tissue. Light green dots represent the RNA editing levels of each RNA editing sites in subcutaneous adipose tissue.

Supplementary Figure 2 RNA editing events in abdominal and subcutaneous adipose tissue of PCOS and control patients. **(A)** The differences in the number of editing sites between the abdominal and subcutaneous adipose tissue of PCOS and control patients. **(B)**The differences in the number of editing genes between the abdominal and subcutaneous adipose tissue of PCOS and control patients. **(C)** Proportion of detected RNA editing sites with known annotation in the REDIportal V2.0 database. Overlap, editing sites of genes in same regions but different chains.

Supplementary Figure 3 RNA editing sites in Ess and Noness genes. **(A)** Editing sites numbers of Ess and Noness genes. **(B)** Editing sites numbers of Ess and Noness genes in nonsynonymous genes. **(C)** The editing frequency of Ess and Noness genes. Between different groups. E, Essential; NE, Non‐essential.

Supplementary Figure 4 RNA editing frequency in abdominal and subcutaneous adipose tissue of PCOS and control patients. **(A)** RNA editing frequency in abdominal and subcutaneous adipose tissue of PCOS and control patients. **(B)** Nonsynonymous and synonymous editing frequency in abdominal and subcutaneous adipose tissue of PCOS and control patients.

Supplementary Figure 5 The intersection of nonsynonymous editing genes between different groups. This Venn diagram represents the intersection of all nonsynonymous editing genes between the abdominal and subcutaneous adipose tissue of PCOS and control patients.

Supplementary Figure 6 GO enrichment analysis of the genes containing tissue-specific RNA editing. Dot plot of the enriched GO Biological Progress terms of genes **(A)** only edited in abdominal adipose tissue not in subcutaneous adipose tissue of PCOS patients and **(B)** only edited in subcutaneous adipose tissue not in abdominal adipose tissue of PCOS patients. Dot color indicates the statistical significance of the enrichment (adjusted p-value); dot size represents the count of genes annotated to each term.

Supplementary Figure 7 PLS-DA analysis of the RNA sequencing data of PCOS and control groups.

Supplementary Figure 8 RNA sequencing of abdominal adipose tissue of PCOS and control patients. **(A)** Heatmap of the differentially expressed genes. **(B)** Dot plot of the enriched GO Biological Progress terms of the genes upregulated expression in abdominal adipose tissue of PCOS compared with the control group. Dot color indicates the statistical significance of the enrichment (adjusted p-value); dot size represents the count of genes annotated to each term.

Supplementary Figure 9 RNA sequencing of abdominal and subcutaneous adipose tissue of PCOS patients. **(A)** Heatmap of the differentially expressed genes. **(B)** Dot plot of the enriched GO Biological Progress terms of the genes upregulated in subcutaneous adipose tissue of PCOS compared with the abdominal adipose tissue of PCOS. **(C)** Dot plot of the enriched GO Biological Progress terms of the genes downregulated in subcutaneous adipose tissue of PCOS compared with the abdominal adipose tissue of PCOS. Dot color indicates the statistical significance of the enrichment (adjusted p-value); dot size represents the count of genes annotated to each term.

Supplementary Figure 10 The correlation between the RNA editing level and RNA expression of the differentially expressed genes in subcutaneous adipose tissue of PCOS and control patients.

Supplementary Figure 11 IGV visualization RNA editing sites in RNA-seq data and the same regions covered by DNA-seq data. **(A)** ATM and **(B)** MALT1 editing sites visualized by IGV. Top is the mapped reads of WGS data, middle is the mapped reads of RNA-seq data, and bottom is the RNA expression of the corresponding gene region. Red squares indicate RNA editing sites and red lines indicate the RNA expression levels of corresponding RNA editing sites.

Supplementary Table 1 Clinical information of the participants.

Supplementary Table 2 List of nonsynonymous editing genes in different groups.

Supplementary Table 3 Genes differentially expressed between subcutaneous adipose tissue of PCOS and control patients.

Supplementary Table 4 Genes differentially expressed between abdominal adipose tissue of PCOS and control patients.

Supplementary Table 5 Genes differentially expressed between abdominal and subcutaneous adipose tissue of PCOS patients.
