# Supplementary figures and images for: RNA editing landscape of adipose tissue in polycystic ovary syndrome provides insight into the obesity-related immune responses
Source: Front Endocrinol (Lausanne). 2024 Jun 24;15:1379293. doi: 10.3389/fendo.2024.1379293 (PMC11229675; doi:10.3389/fendo.2024.1379293)

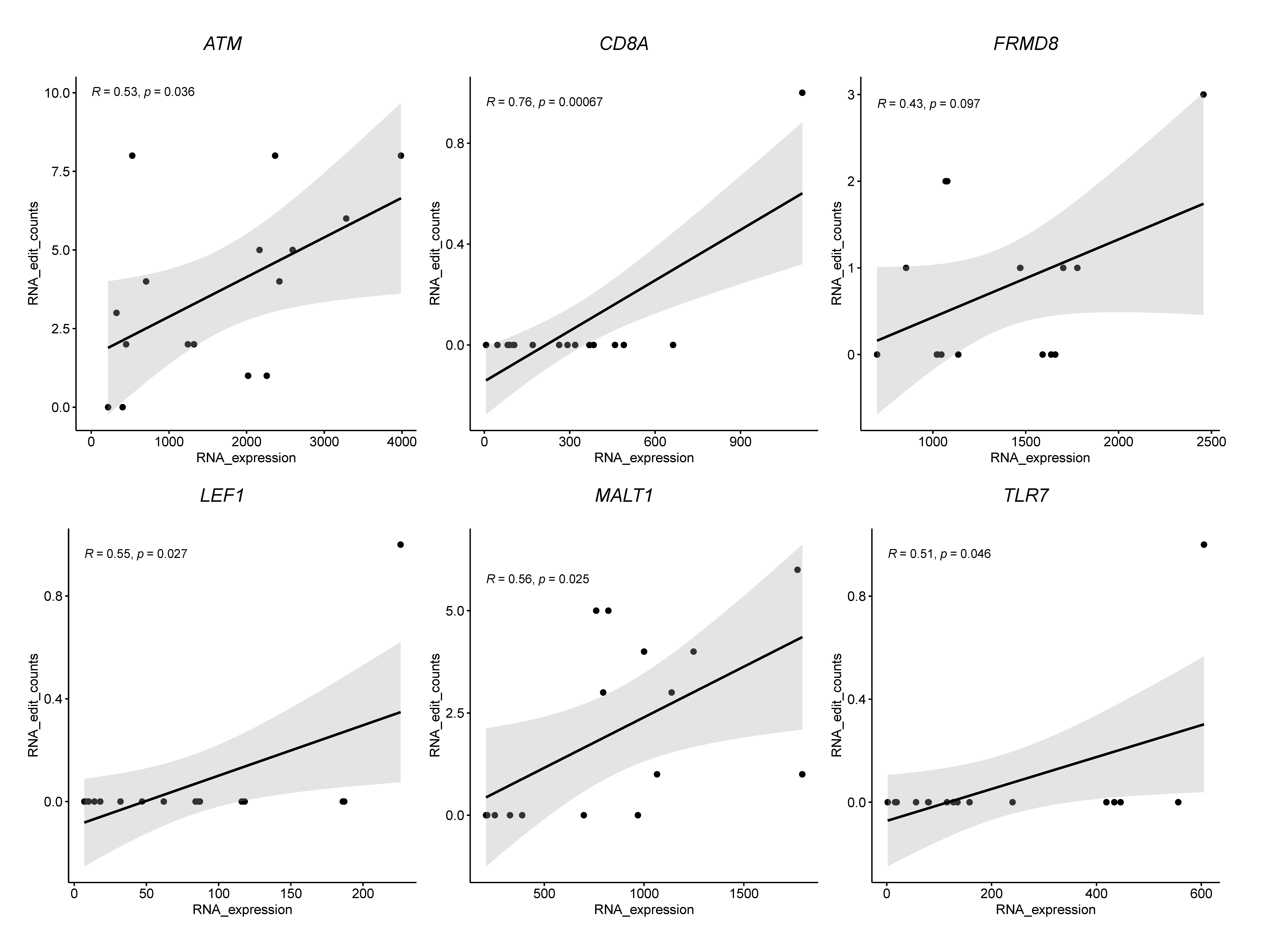

Supplement: Supplementary Figure 1 — Mapping of the RNA editing sites and the DNA sequencing data of each individual visualizing by Circos plot. (A) control samples. From left to right is control 1 to 3, respectively. (B) PCOS samples. From left to right and up to down is PCOS 1 to 5, respectively. Pink dots represent the quality score of each SNV from DNA sequencing data. Orange dots represent the RNA editing levels of each RNA editing sites in abdominal adipose tissue. Light green dots represent the RNA editing levels of each RNA editing sites in subcutaneous adipose tissue. [file DataSheet_1.zip › Supplementary Figure 10.tiff]

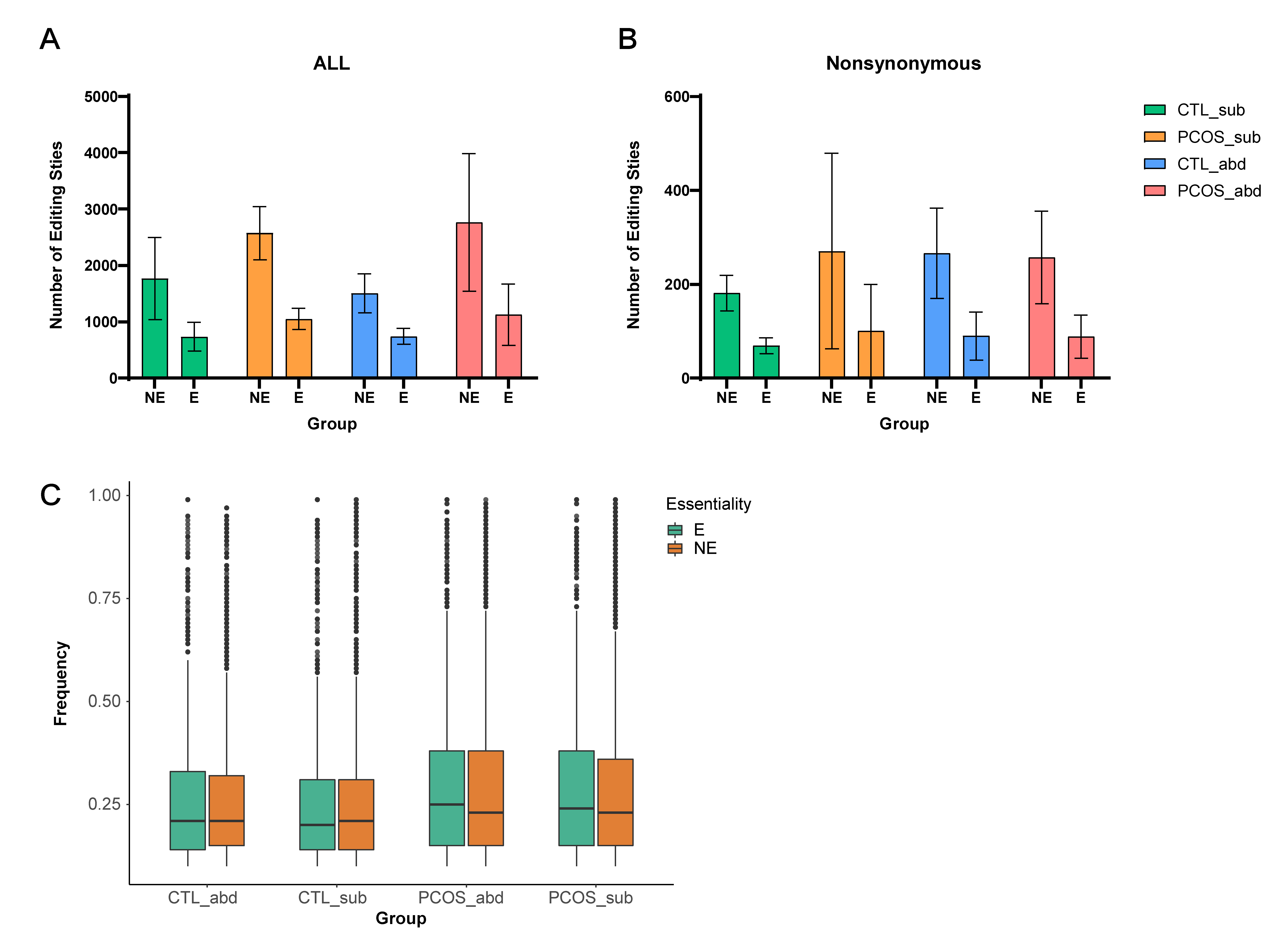

Supplement: Supplementary Figure 1 — Mapping of the RNA editing sites and the DNA sequencing data of each individual visualizing by Circos plot. (A) control samples. From left to right is control 1 to 3, respectively. (B) PCOS samples. From left to right and up to down is PCOS 1 to 5, respectively. Pink dots represent the quality score of each SNV from DNA sequencing data. Orange dots represent the RNA editing levels of each RNA editing sites in abdominal adipose tissue. Light green dots represent the RNA editing levels of each RNA editing sites in subcutaneous adipose tissue. [file DataSheet_1.zip › Supplementary Figure 3.tiff]

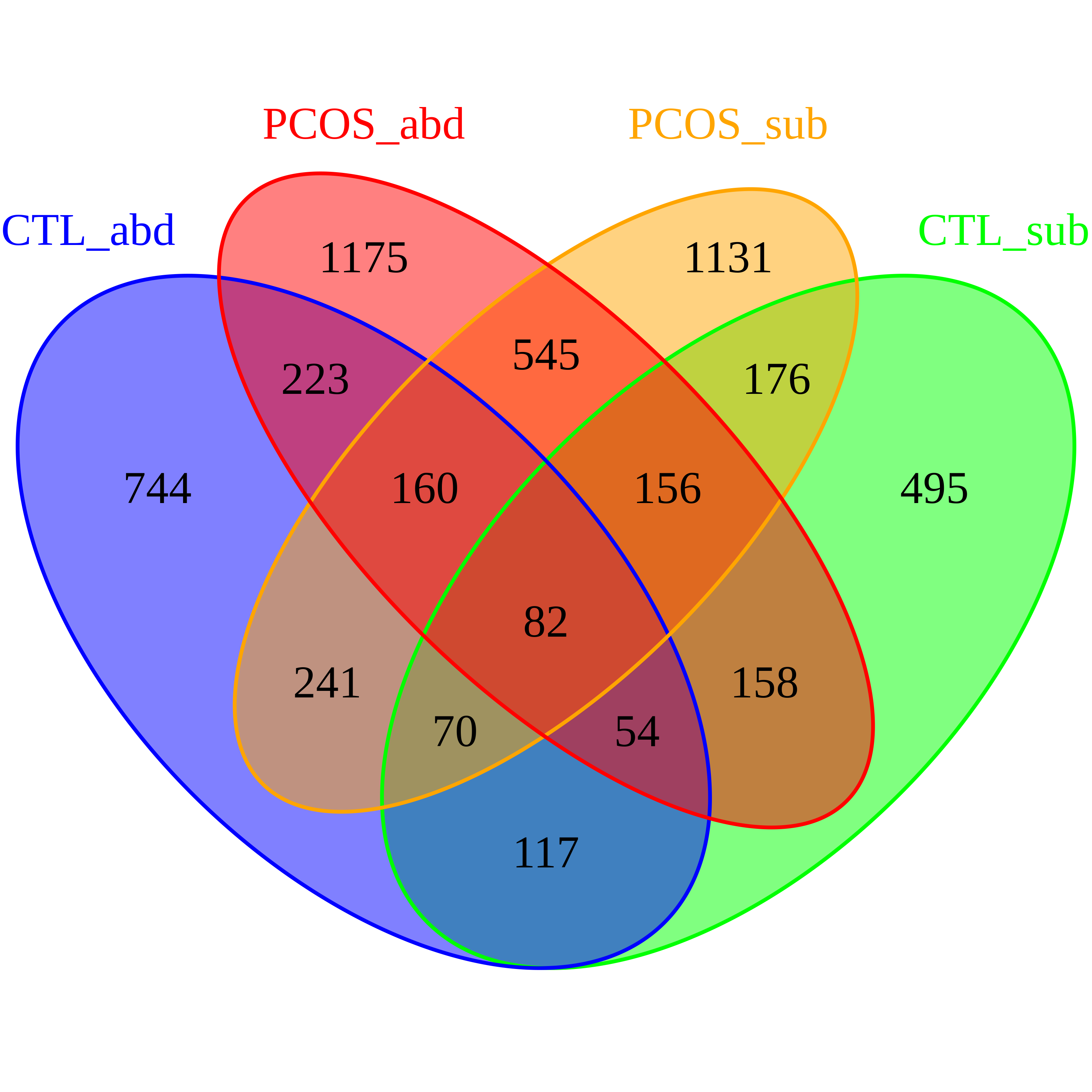

Supplement: Supplementary Figure 1 — Mapping of the RNA editing sites and the DNA sequencing data of each individual visualizing by Circos plot. (A) control samples. From left to right is control 1 to 3, respectively. (B) PCOS samples. From left to right and up to down is PCOS 1 to 5, respectively. Pink dots represent the quality score of each SNV from DNA sequencing data. Orange dots represent the RNA editing levels of each RNA editing sites in abdominal adipose tissue. Light green dots represent the RNA editing levels of each RNA editing sites in subcutaneous adipose tissue. [file DataSheet_1.zip › Supplementary Figure 5.png]

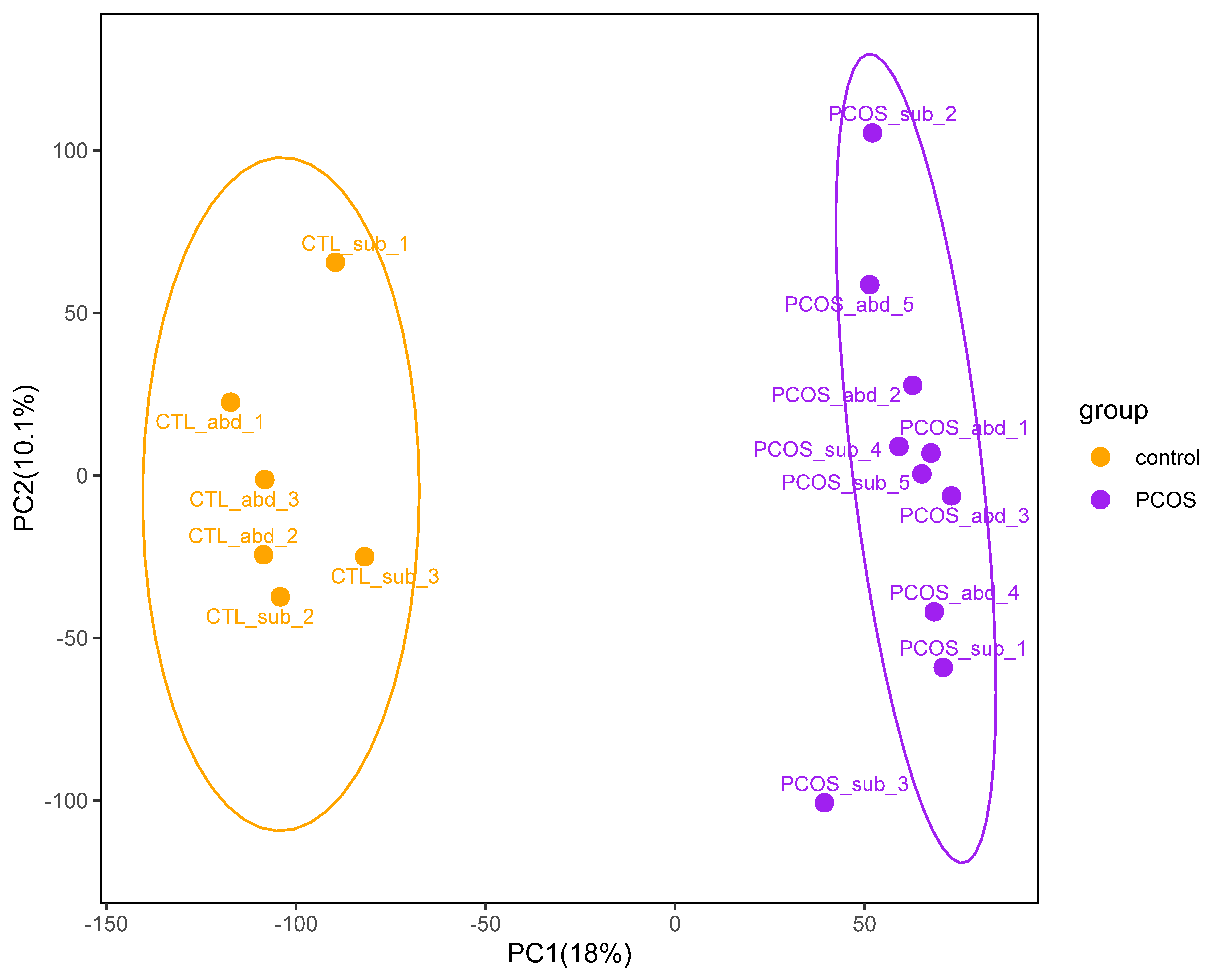

Supplement: Supplementary Figure 1 — Mapping of the RNA editing sites and the DNA sequencing data of each individual visualizing by Circos plot. (A) control samples. From left to right is control 1 to 3, respectively. (B) PCOS samples. From left to right and up to down is PCOS 1 to 5, respectively. Pink dots represent the quality score of each SNV from DNA sequencing data. Orange dots represent the RNA editing levels of each RNA editing sites in abdominal adipose tissue. Light green dots represent the RNA editing levels of each RNA editing sites in subcutaneous adipose tissue. [file DataSheet_1.zip › Supplementary Figure 7.tif]
